# The Biphasic Effects of Oxidized-Low Density Lipoprotein on the Vasculogenic Function of Endothelial Progenitor Cells

**DOI:** 10.1371/journal.pone.0123971

**Published:** 2015-05-27

**Authors:** Feng-Yen Lin, Nai-Wen Tsao, Chun-Ming Shih, Yi-Wen Lin, Jong-Shiua Yeh, Jaw-Wen Chen, Hironori Nakagami, Ryuichi Morishita, Tatsuya Sawamura, Chun-Yao Huang

**Affiliations:** 1 Department of Internal Medicine, School of Medicine, College of Medicine, Taipei Medical University, Taipei, Taiwan; 2 Division of Cardiology, Department of Internal Medicine and Cardiovascular Research Center, Taipei Medical University Hospital, Taipei, Taiwan; 3 Division of Cardiovascular Surgery, Taipei Medical University Hospital, Taipei, Taiwan; 4 Division of Cardiovascular Medicine, Department of Internal Medicine, Wan Fang Hospital, Taipei Medical University, Taipei, Taiwan; 5 Department of Medical Education and Research, Taipei Veterans General Hospital, Taipei, Taiwan; 6 Division of Vascular Medicine and Epigenetics, Osaka University, Osaka, Japan; 7 Department of Clinical Gene Therapy, Osaka University, Osaka, Japan; 8 Department of Bioscience, National Cardiovascular Center Research Institute, Osaka, Japan; Centro Cardiologico Monzino, ITALY

## Abstract

Late-outgrowth endothelial progenitor cells (EPCs) are stress-resistant and responsible for reparative functions in the cardiovascular system. Oxidized-LDL (oxLDL) plays a critical role in cardiovascular disease pathogenesis. However, it is largely unknown what the impacts of oxLDL are on late-outgrowth EPCs. This study aimed to investigate the concentration-related effects of oxLDL on EPC functions and related angiogenesis, *in vitro* and *in vivo*. In this study, early and late-outgrowth EPCs were generated from circulating human mononuclear cells. oxLDL may regulate EPC vasculogenic function via the lectin-like oxidized low-density lipoprotein receptor-1 (LOX-1). Lower concentrations (5 μg/mL) of oxLDL can potentiate EPC tube formation *in vitro* and *in vivo* by activating eNOS mechanisms, which are mediated by p38 MAPK- and SAPK/JNK-related pathways. Higher concentrations of oxLDL (10-50 μg/mL) impaired EPC function via the activation of nicotinamide adenine dinucleotide phosphate (NADPH) oxidase pathways and consequent inhibition of eNOS activity, which could be reversed by anti-oxidants (diphenylene iodonium and apocynin) and gp91^*phox*^ siRNA. In conclusion, oxLDL has concentration-dependent biphasic effects on human late-outgrowth EPC tube formation *in vitro* and *in vivo*.

## Introduction

Lipid metabolic disorder is one of the most important and common risk factors for atherosclerosis, impaired angiogenesis [1–3] and the prevalence of coronary artery disease [4–6]. Various mechanisms can lead to dyslipidemia. Lipid oxidation, especially oxidized-low density lipoprotein (oxLDL) can enhance intra-atherosclerotic plaque inflammation [7] and instability [8,9], promoting the formation of early and full-brown atherosclerosis [10–12] resulting in acute coronary syndrome and depending on its severity [13,14], impaired angiogenesis, vasculogenesis and collateral vessel formation in ischemic tissue [15]. In addition, serum oxLDL levels have been demonstrated to predict long term survival in the elderly [16].

Circulating endothelial progenitor cells (EPCs) are considered to have several reparative functions on impaired endothelium and are also involved in neo-vasculogenesis in ischemic tissue as well as tumor microenvironments [17–19]. Previous data has shown that circulating EPCs may have special cellular machinery that is resistant to various types of stress, including oxidative stress [20], which may enable EPCs to participate in tissue repair. In addition, studies on EPC-based cell therapy for full brown cardiovascular disease have been performed intensively. However, some studies have still failed to demonstrate the involvement of EPCs in the regeneration process of ischemic tissue and a growing body of evidence suggests the contrary [21,22]. A study conducted by David Ingram et al. used more purified EPCs and clonogenic assays to test the effects of oxidative stress on EPC function. This study demonstrated that EPCs, unlike stem cells, are still vulnerable to environmental risk factors, particularly oxidative stress, which is abundant in the reparative ischemic environment [23].

Previous studies have suggested that oxLDL may have detrimental effects on EPCs [24–28]. However, a limitation of most of these studies is their use of early EPCs—which mainly play paracrine functions, unlike the adherent late-outgrowth EPCs, which are demonstrated to be further involved and incorporated into vascular repair and neovasculogenesis in ischemic tissue [29]. In addition, most of these studies used concentrations of oxLDL that (more than 50 μg/mL) were much higher than the physiological serum concentrations of healthy (5.7–14.8 μg/mL) and diseased human beings (18.8–20.2 μg/mL in DM and coronary artery disease) [30–35]. Therefore, the behaviors of EPCs have not been thoroughly investigated under physiological concentrations of oxLDL

Knowing the potential role of EPCs in regenerative medicine and that dyslipidemia is a main risk factor for cardiovascular disease, we designed this study using late-outgrowth EPCs treated with oxLDL within physiological serum concentrations (0.1–50 μg/mL). We investigated the potential impacts and underlying mechanisms of oxLDL on EPC vasculogenesis, which could help further advance EPC-based regenerative treatments in patients with dyslipidemia.

## Materials and Methods

### *In Vitro* Study

#### Reagents and Antibodies

Rabbit anti-SR-A, rabbit anti-SR-B1, and goat anti-hCD34 antibodies were purchased from Santa Cruz Co. (Santa Cruz, CA, USA). LOX-1 blocking antibody was a kind gift from Dr. Tatsuya Sawamura of Japan. The rabbit anti-phospho-eNOS and total-eNOS antibodies were purchased from Millipore Co. (North Billerica, MA, USA). The anti-Akt and anti-MAPKs antibodies were all purchased from Cell Signaling Co. (Danvers, MA, USA). All chemical reagents, including L-arginine methyl ester hydrochloride (L-NAME), S-nitroso-N-acetylpenicillamine (SNAP), N-acetylcysteine (NAC), diphenyleneiodonium (DPI), apocynin (APO), LY294002, PD98059, SB203580, SP600125, and the Rac1 inhibitor were purchased from Calbiochem-Merck (KGaA, Darmstadt, Germany). The gp91^phox^ siRNA was purchased from Thermo Scientific Inc. (San Jose, CA, USA).

#### Preparation of Oxidized Low Density Lipoprotein

The LDL fraction of human serum was isolated and characterized as previously described [36,37]. Briefly, plasma was taken from blood withdrawn into 0.38% sodium citrate from healthy young adult males. The major lipoprotein classes of LDL (d = 1.019–1.063 g/mL) were prepared by sequential ultracentrifugation. Density was adjusted by using solid NaBr and extensive dialysis at 4°C for 24 hours against phosphate-buffered saline (PBS, 5 mM phosphate buffer and 125 mM NaCl, pH 7.4). LDL was oxidized by dialysis for 24 hours at 37°C against 10 μM CuSO_4_ in PBS, then oxLDL was dialyzed for 24 hours at 4°C against PBS containing 0.3 μM EDTA. The extracted oxLDL was stored in the dark at -80°C until use. In these experiments, the extent of oxidation was monitored by measurement of thiobarbituric acidreactive substance (TBARS) and horizontal electrophoresis. To avoid endotoxin contamination, isolated lipoproteins were acquired from healthy volunteers (no illness), in a sterile environment. Isolated lipoprotein samples were also grown on bacterial culture discs to exclude the possibility of bacterial contamination. In this study, we also maintained the 7.5 ± 1.5 μM of MDA in the oxidation state of per microgram of native LDL, and 90.0 ± 10.5 μM of MDA in the oxidation state of per microgram of oxLDL. The oxidation state of oxLDL is 7–11 times to the nLDL. Only oxLDL prepared within 7 days was used.

#### EPC Isolation and Cultivation

Total mononuclear cells (MNCs) were isolated from 40 ml of peripheral blood from healthy young male volunteers using density gradient centrifugation with Histopaq-1077 (density 1.077 g/mL; Sigma). The Taipei Medical University-Institutional Review Board approved this study (IRB No.: CRC-04-10-04 and No.: 201302008), and all participants provided their written informed consent to participate in this study. MNCs (1x10^7^ cells) were plated in 2 ml of endothelial growth medium (EGM-2 MV; Cambrex, Charles, IA, USA) with supplements (hydrocortisone, R3-insulin-like growth factor 1, human vascular endothelial growth factor, human fibroblast growth factor, gentamicin, amphotericin B, vitamin C, and 20% fetal bovine serum) on fibronectin-coated six-well plates at 37°C in a 5% CO2 incubator. The cultures were observed daily, and after 4 days of culture, the media was changed and nonadherent cells were removed. EPCs that attached early had a spindle shape and were elongated. Thereafter, media was replaced every 3 days, and each colony/cluster was observed. A certain number of early EPCs continued to grow into colonies of late EPCs, which emerged 2–4 weeks after the start of MNC culture. The late EPCs exhibited “cobblestone” morphology and a monolayer growth pattern typical of mature endothelial cells at confluence. The late EPC-derived EC outgrowth population was also characterized using immunofluorescent staining for the expression of lectin, VE-cadherin, von Willebrand factor (vWF), CD31 (platelet/endothelial cell adhesion molecule; PE-CAM), CD34, kinase insert domain receptor (KDR)/VEGF receptor 2, CD133 and the endocytic portion of Dil-acLDL. The vascular smooth muscle cell markers (αSMA, CALP, SM-MHC) and leukocyte marker (CD45) were undetectable. The fluorescent images were recorded using a laser scanning confocal microscope (S1 Fig).

#### Measurement of Cytotoxicity by MTT Assay

The cytotoxicity of oxLDL was analyzed using the 3-(4,5-dimethylthiazol-2-yl)-2,5-diphenyltetrazolium bromide (MTT) assay. EPCs (2x10^4^ cells) were grown in 96-well plates and incubated with oxLDL at 0–100 μg/mL for 12 or 24 hours. Subsequently, MTT (0.5 μg/mL) was applied to cells for 4 h to allow the conversion of MTT into formazan crystals. After washing with PBS, the cells were lysed with dimethyl sulfoxide, and the absorbance was read at 530 nm with a DIAS Microplate Reader (Dynex Technologies, Chantilly, VA, USA).

#### EPC Tube Formation Assay

The tube formation assay was performed on EPCs to assess angiogenic capacity, which is believed to be important for new vessel formation. The *in vitro* tube formation assay was performed using the Angiogenesis Assay Kit (Chemicon, CA, USA)[38] according to the manufacturer’s protocol. Briefly, ECMatrix gel solution was thawed at 4°C overnight, mixed with ECMatrix diluent buffer, and placed in a 96-well plate at 37°C for 1 hour to allow the matrix solution to solidify. EPCs were treated with oxLDL for 24 hours and then harvested. A total of 10^4^ cells were placed on the matrix solution, and the samples were incubated at 37°C for 12 hours. Tubule formation was inspected under an inverted light microscope, four representative fields were taken. The average of the total area of complete tubes formed by cells was compared using the Image-Pro Plus computer software.

#### Knockdown of Gene Expression by Silencing RNA

Knockdown of gp91^phox^ gene expression was performed by transfection of EPCs with silencing RNA (siRNA) using GenMute siRNA Transfection Reagent (SignaGen Lab., Gaithersburg, MD, USA). Cells (3x10^6^) were suspended in 2.5 ml of serum-free medium and 25-μM aliquots of a gp91^phox^ siRNA duplex mixture (Catalog CYBB-HSS 102523, CYBB-HSS 175761, and CYBB-HSS 102525; Invitrogen, Carlsbad, CA, USA) were transfected according to the manufacturer’s instructions. Cells were seeded into 6-well plates immediately post-transfection, and experiments were done 24 hours later.

#### Western Blot Analysis

Total cell lysate and membrane proteins were processed according to previous reports [39]. The protein concentration in the supernatants was measured using a Bio-Rad protein determination kit (Bio-Rad, CA, USA). The supernatants were subjected to 8% SDS-PAGE and transferred for 1 hour at room temperature to polyvinylidene difluoride membranes. The membranes were treated for 1 hour at room temperature with PBS containing 0.05% Tween-20 and 2% skimmed milk and incubated separately for 1 hour at room temperature with primary antibodies. The membranes were then incubated with horseradish peroxidase-conjugated IgG. Immunodetection was performed using a chemiluminescence reagent and with exposure to Biomax MR Film (Kodak, NY, USA).

#### Electron Spin Resonance Spectroscopy (ESRS)

Spin-probe colloid Fe(DETC)2 (Noxygen) was used as a probe to detect the NO production in EPCs and was performed as previously described [40,41]. In brief, 200 μL of EPC-cultured medium was mixed with 400 μL of colloid Fe(DETC)2, followed by incubation for 1 hour at 37°C. The samples were recorded on a ESRS (model: EMX-6/1, Bruker BioSpin, San Antonio, TX, USA) with the following instrumental settings: center field 3295.0 G, sweep width 100.0 G, static field 3415.0 G, power of microwave 2.0 mW, microwave frequency 9.8 GHz, modulation amplitude 10.0 G, modulation frequency 100.0 GHz, 1024 points resolution in X, sweep time 10.5 seconds and number of X-scans 5.

### *In Vivo* Study

#### Preparation of Animals

All animals were treated according to protocols approved by the Institutional Animal Care and Use Committee (IACUC) of the Taipei Medical University, Taipei, Taiwan (admission No.: LAC 101–0127). Twenty-seven adult male NOD SCID mice (6-week old) were purchased from the Jackson Laboratory (Bar Harbor, ME, USA). Mice were kept in microisolation cages on a 12-h day/night cycle and fed a commercial mouse chow diet (Scientific Diet Services, Essex, UK) with water *ad libitum*. Experimental procedures and animal care conformed to the ‘‘Guide for the Care and Use of Laboratory Animals” published by the US National Institutes of Health (NIH Publication No. 85–23, revised 1996).

#### Production of Neoangiogenesis Gel

Production of neoangiogenesis gel was modified from a previous study [42]. The neoangiogenesis gel solution contained 1.5 mg/mL of rat-tail type 1 collagen, 90 μg/mL human plasma fibronectin, 25 mM Hepes, 1.5 mg/mL NaHCO_3_, and EGM-2 medium and was stored at 4°C until use. Isolated EPCs were suspended in neoangiogenesis gel solution and then pipetted into 12-transwell plates and warmed to 37°C for 10 minutes to allow polymerization of the neoangiogenesis gel. In some experiments, neoangiogenesis/EPC gels contained oxLDL with or without 100 μM of APO, 30 μM of DPI, 10 μM of SP600125, 10 μM of SB203580 or 10 μg/mL of LOX-1 blocking antibody. After polymerization was completed, warmed EGM-2 was added to cover the solidified gels, and the gels were cultured at 37°C in 5% CO_2_ for 24 hours. For implantation, neoangiogenesis/EPC gels were harvested from the transwell plates and approximately 1x1x0.5 cm gel segments were dissected.

#### Study Protocol

The animals were anesthetized using intraperitoneal injection of Xylocaine (2 mg/kg of BW) plus Zoletil (containing a dissociative anesthetic, Tiletamine/ Zolazepam at a ratio of 1:1; 5 mg/kg of BW). The neoangiogenesis/EPC gels were subcutaneously implanted into bluntly dissected subcutaneous pouches in the backs of the NOD SCID mice. The wounds were closed with skin sutures. At the time indicated (21 days), the mice were anesthetized and the implanted constructs were harvested. The implanted constructs were examined in 5-μμm frozen sections, and vascular-like density was determined using a goat monoclonal antibody directed against human CD31 (Santa Cruz, CA, USA). Four representative fields were taken, and the average of the total area of complete tubes formed by the cells was compared using the Image-Pro Plus computer software.

#### Statistical Analyses

Values are expressed as the mean ± SEM. Statistical evaluation was performed using Student’s t test and one- or two-way ANOVA followed by a Dunnett’s test. A probability value of p<0.05 was considered significant.

## Results

### OxLDL with Biphasic Effects in EPC Capacity for Tube Formation

**Fig 1:**
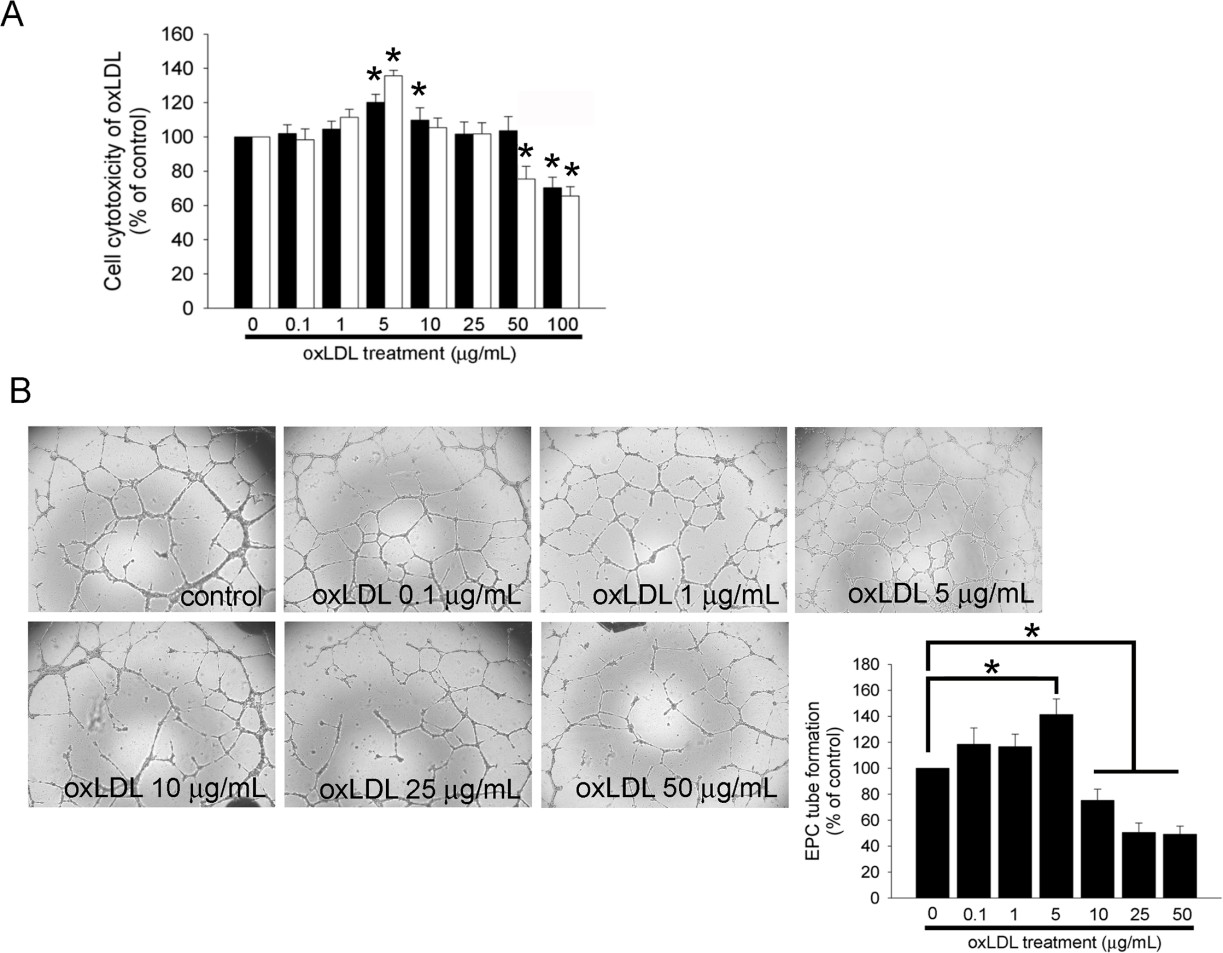
The effects of oxLDL on cell cytotoxicity and tube formation. (A) After treatment of EPCs with 0–100 μg/mL of oxLDL for 12 (▓) or 24 (□) hours, an MTT assay was performed, and the absorbance was recorded using a microplate reader. (B) EPCs were treated for 24 hours with 0–50 μg/mL oxLDL. An *in vitro* angiogenesis assay was used with ECMatrix gel to investigate the effect of oxLDL on EPC neovascularization. Representative photos of *in vitro* angiogenesis are shown. Data are expressed as the mean ± SEM of nine experiments performed in triplicate.*p < 0.05 was compared to a control group with the same time treatment and considered significant.

An MTT assay was performed to analyze cell viability and cytotoxicity of oxLDL. EPCs were treated with 0.1–100 μg/mL of oxLDL for 12 or 24 hours. The results showed that treatment with 5 μg/mL of oxLDL both for 12 and 24 hours as well as 10 μg/mL of oxLDL for 12 hours increased cell viability. In contrast, treatment with 50 μg/mL of oxLDL for 24 hours or 100 μg/mL of oxLDL both for 12 and 24 hours reduced cell viability as well as increased cell cytotoxicity in a dose-dependent manner (Fig 1A). To explore the potential effects of oxLDL on EPC angiogenesis, a tube formation assay was performed. It has been shown that EPCs can successfully initiate capillary network formation on Matrigel [43]. After 24 hours of culture in 5 μg/mL oxLDL, the functional capacity for tube formation of EPCs on ECMatrix gel significantly increased compared to the control group (141.3 ± 12.0% of control). Interestingly, treatment of EPCs with oxLDL at 10, 25, or 50 μg/mL for 24 hours significantly decreased cell tube formation (75.3 ± 8.6%, or 50.6 ± 7.2%, or 49.2 ± 6.3% of control, respectively) compared to the control group. These results indicate that low concentrations (approximately 5 μg/mL) of oxLDL potentially increase the tube formation capacity of EPCs, whereas higher concentrations (more than 10 μg/mL) of oxLDL induce cell cytotoxicity and impair EPC tube formation.

### OxLDL Affects Tube Formation Capacity Mediated by LOX-1 and Enhances LOX-1 Expression in EPCs

**Fig 2:**
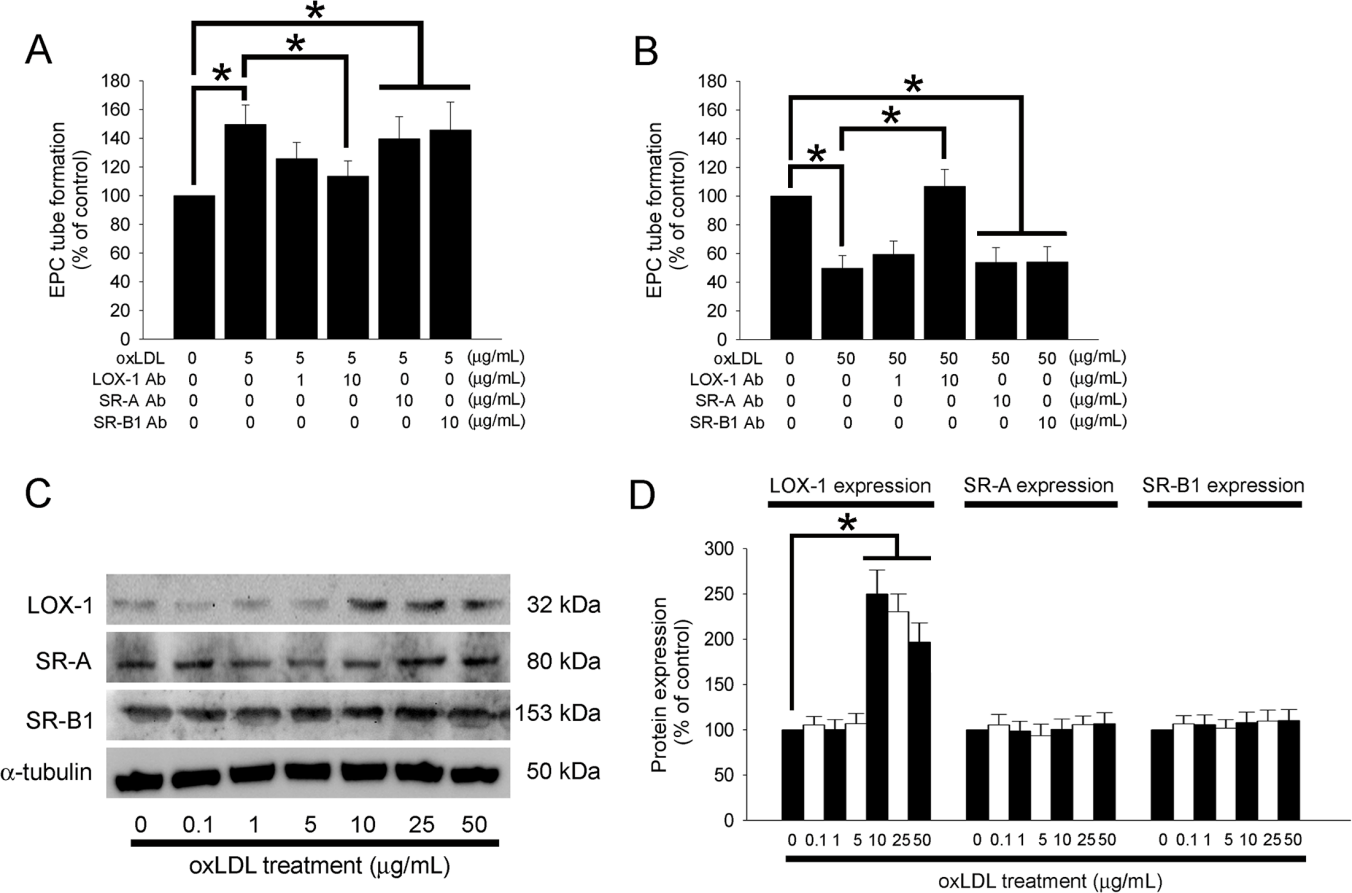
Effects of oxLDL on tube formation in EPCs is mediated by LOX-1. (A and B) EPCs were pretreated with anti-SR-A, anti-SR-B1, or LOX-1 blocking antibodies for 1 hour prior to oxLDL treatment. The effect of oxLDL on EPC neovascularization was analyzed using an *in vitro* angiogenesis assay. (C) EPCs were treated for 12 hours with 0–50 μg/mL oxLDL, and membrane LOX-1, SR-A, and SR-B1 were analyzed by Western blot. α-tubulin protein levels were used as a loading control. (D) The graph shows the quantification of LOX-1, SR-A, and SR-B1 density in oxLDL-treated EPCs. Data are expressed as the mean ± SEM of three experiments. *p < 0.05 was compared to the control group in the same time treatment and considered significant.

LOX-1, SR-A and SR-B1 were originally identified on the membrane of vascular endothelial cells and EPCs. Therefore, we investigated whether the potential effects of oxLDL on EPC angiogenesis are mediated by these membrane receptors. Neovascularization was analyzed using an *in vitro* angiogenesis assay in EPCs incubated with rabbit anti-SR-A, rabbit anti-SR-B1, or LOX-1 blocking antibody (10 μg/mL) for 1 hour followed by 5 or 50 μg/mL of oxLDL treatment. Fig 2A shows that 10 μg/mL of LOX-1 blocking antibody, but not SR-A or SR-B1 blocked the promotion of EPC tube formation capacity under 5 μg/mL of oxLDL treatment. Similar to Fig 1A, addition of 10 μg/mL of LOX-1 blocking antibody may have significantly reduced the impaired tube formation capacity of EPCs treated with 50 μg/mL oxLDL. As a negative control in the competition assay, a nonspecific IgG2α isotype antibody was substituted for the specific antibodies (data not shown). Additionally, we also analyzed membrane LOX-1, SR-A, and SR-B1 production with Western blot of oxLDL-treated EPCs. As shown in Fig 2C, an oxLDL concentration of more than 10 μg/mL may elevate LOX-1 expression in EPCs; in contrast, neither SR-A nor SR-B1 was affected by oxLDL treatment.

These results demonstrate that the increased neovascularization capacity of EPCs with 5 μg/mL oxLDL and the impaired neovascularization capacity of EPCs with 50 μg/mL oxLDL- are both mediated by LOX-1, but not SR-A or SR-B1.

### The PI3K/Akt- and NO-related Pathways Contribute to the Effects of OxLDL on EPC Tube Formation

**Fig 3:**
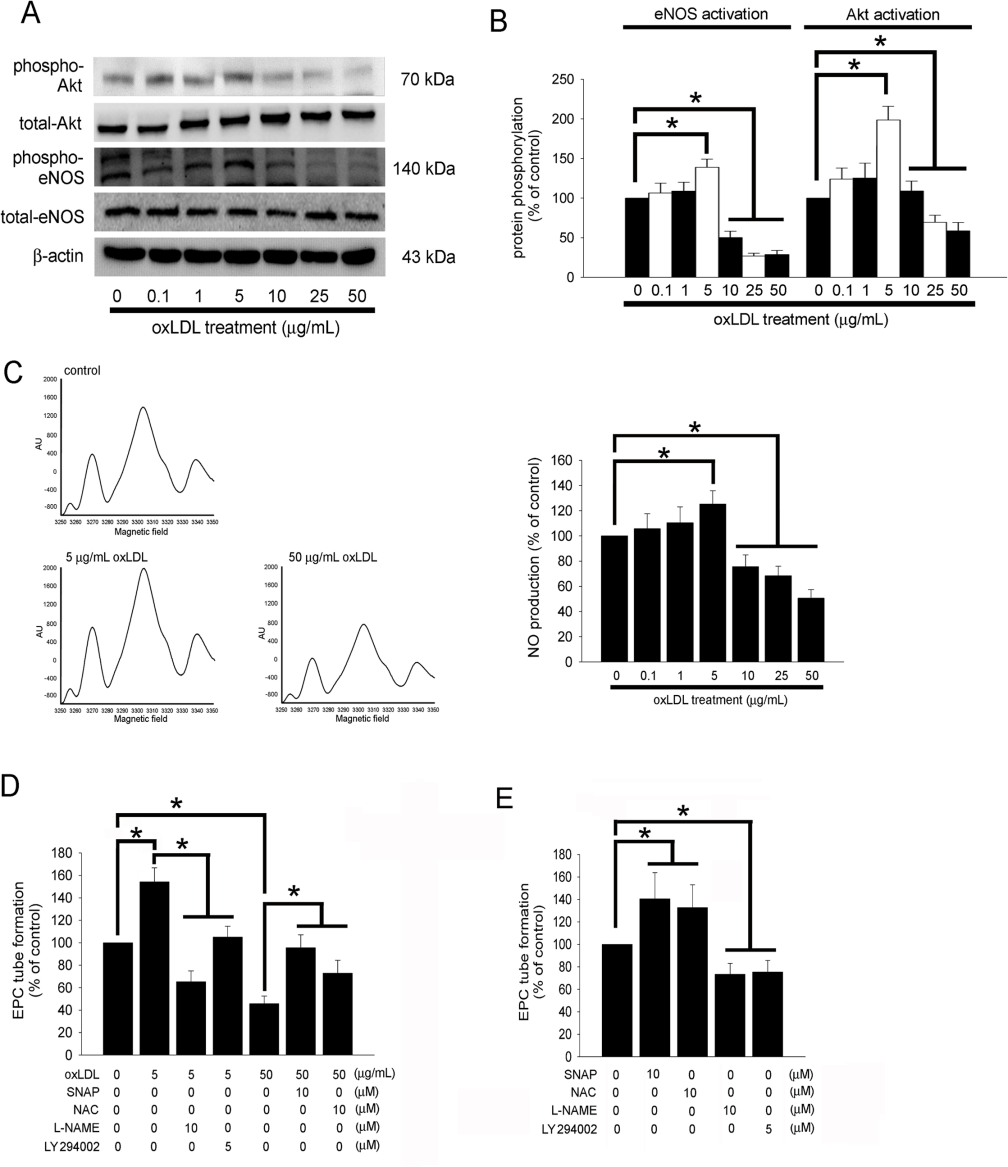
The PI3K/Akt- and NO-related pathways contribute to EPC tube formation affected by oxLDL. (A and B) EPCs were treated for 2 hours with 0–50 μg/mL oxLDL, then eNOS and Akt activation (phosphorylation) was analyzed by Western blot. Total eNOS, Akt, and β-actin protein levels were used as loading controls. The graphs show the quantitative activation of eNOS (phospho-eNOS/total-eNOS ratio) and Akt (phospho-Akt/total-Akt ratio) density in oxLDL-treated EPCs. (C) EPCs were pretreated with 0–50 μg/mL oxLDL for 24 hours. The NO-containing conditional medium was analyzed using electron spin resonance spectroscopy. Left, Representative ESR spectra of the NO-Fe(DETC)2 signal in EPCs treated with 0, 5 or 50 μg/mL oxLDL. Right, increasing concentrations of oxLDL (0–5 μg/mL) in EPCs from healthy subjects stimulated NO production. In contrast, 10–50 μg/mL oxLDL had decreasing production of NO observed (n = 5). (D) EPCs were pretreated with L-NAME or LY294002 for 1 hour prior to treatment with 5 μg/mL oxLDL for 24 hours, or pretreated with SNAP or NAC for 1 hour prior to treatment with 50 μg/mL oxLDL for 24 hours. (E) EPCs were treated with SNAP, NAC, L-NAME or LY294002 alone for 24 hours. *In vitro* angiogenesis was assayed using ECMatrix gel. Data are expressed as the mean ± SEM of three experiments performed in triplicate. *p < 0.05 was considered significant.

We have previously shown that phosphatidylinositol-3 kinase (PI3K)/Akt- and NO-related mechanisms could be involved in the angiogenesis capacity of high glucose-stimulated EPCs [43]. In this study, we investigated whether PI3K/Akt and NO regulate tube formation in oxLDL-treated EPCs. After cultured EPCs were incubated for 2 hours with 5 μg/mL of oxLDL, Akt phosphorylation and eNOS phosphorylation at Ser^1,177^ were significantly increased (Fig 3A). Furthermore, Akt phosphorylation and eNOS phosphorylation at Ser^1,177^ were significantly decreased in EPCs incubated with more than 10 μg/mL oxLDL. The conditional medium containing NO was analyzed using electron spin resonance spectroscopy. In Fig 3C, 5 μg/mL oxLDL treatment may have markedly increased NO production in comparison to the control (no oxLDL treatment) group; however, incubation with 10–50 μg/mL oxLDL may have decreased NO production. Finally, we measured whether PI3K/Akt- and NO-related signaling pathways were involved in the angiogenic capacity of EPCs stimulated with oxLDL. Pretreatment with the NOS inhibitor l-N^g^-nitro-l-arginine methyl ester (L-NAME) or PI3K inhibitor, LY294002 for 1 hour significantly ameliorated the positive effects of 5 μg/mL oxLDL on EPC tube formation capacity (Fig 3D). In contrast, pretreated with 10 μM of NO donors, S-nitroso-N-acetylpenicillamine (SNAP) or N-acetylcysteine (NAC) for 1 hour significantly ameliorated inhibition of tube formation in EPCs stimulated with 50 μg/mL oxLDL for 24 hours. Treatment with SNAP, NAC, L-NAME, or LY294002 for 24 hours had been used the controls (Fig 3E). Therefore, these data indicate that oxLDL protein may affect the capacity of neovascularization (both by promoting and inhibiting) in EPCs by modulating PI3K/Akt- and eNOS-related mechanisms.

### The p38 MAPK- and SAPK/JNK-related Pathways Contribute to EPC Tube Formation Increased by Low OxLDL Concentrations

**Fig 4:**
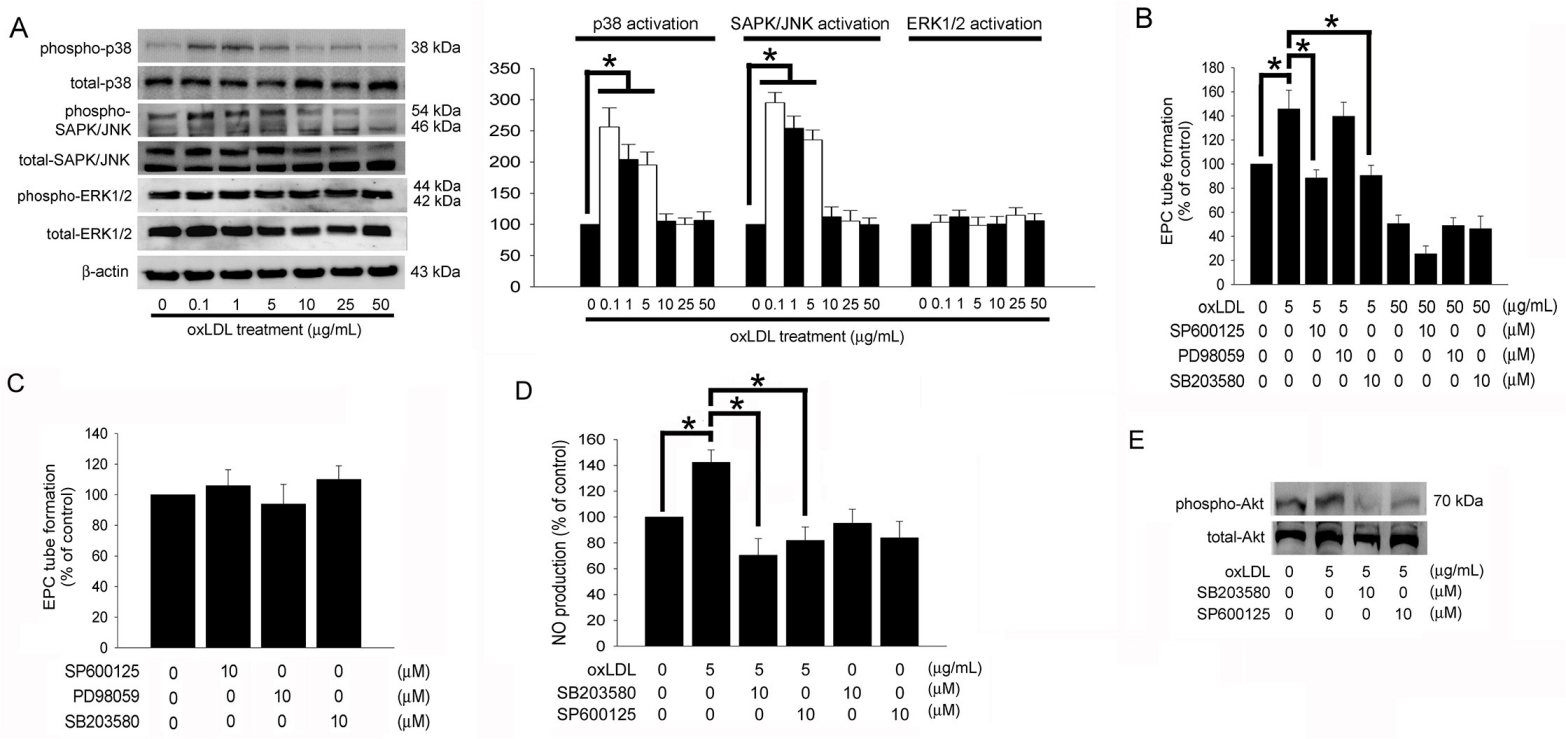
p38 MAPK- and SAPK/JNK-related pathways contribute to increased tube formation in EPCs under low concentrated oxLDL. (A) Following treatment of EPCs with 0–50 μg/mL of oxLDL for 4 hours, the phosphorylation of p38 MAPK, SAPK/JNK and ERK1/2 were analyzed by Western blot. The total p38 MAPK, SAPK/JNK, ERK1/2, and β-actin protein levels were used as loading controls. The graph showed the quantitative activation of p38 MAPK (phospho-p38MAPK/total- p38MAPK ratio), SAPK/JNK (phospho-SAPK/JNK/total-SAPK/JNK ratio), and Akt (phospho-ERK1/2/total-ERK1/2 ratio) density in oxLDL-treated EPCs. (B) EPCs were pretreated with SP600125, PD98059, or SB203580 for 1 hour prior to treatment with 5 or 50 μg/mL oxLDL for 24 hours. (C) Treatment of SP600125, PD98059, or SB203580 alone for 24 hours. *In vitro* angiogenesis was assayed using ECMatrix gel. (D) EPCs were pretreated with SP600125 or SB203580 for 1 hour prior to treatment with 5 μg/mL oxLDL for 24 hours. The NO-containing conditional medium was analyzed using electron spin resonance spectroscopy. Data are expressed as the mean ± SEM of three experiments performed in triplicate. *p < 0.05 was considered significant. (E) EPCs were pretreated with SP600125 or SB203580 for 1 hour prior to treatment with 5 μg/mL oxLDL for 2 hours, subsequently Akt activation (phosphorylation) was analyzed by Western blot. Total Akt protein levels were used as loading controls.

The potential roles of mitogen-activated protein kinase (MAPK)-related mechanisms were also examined. OxLDL markedly induced the phosphorylation of MAPKs, including p38 MAPK and SAPK/JNK (Fig 4A), after exposure to 0.1–5 μg/mL of oxLDL for 4 hours. Interestingly, exposure to 10–50 μg/mL of oxLDL did not change the phosphorylation of p38 MAPK, ERK1/2, and SAPK/JNK in EPCs.

Pretreatment with SP600125 (a SAPK/JNK inhibitor) and SB203580 (a p38 MAPK inhibitor) but not PD98059 (an ERK1/2 inhibitor) for 1 hour may have ameliorated the positive effects of 5 μg/mL oxLDL on EPC tube formation (Fig 4B). Treatment of MAPK inhibitors alone did not affect the tube formation in EPCs (Fig 4C). Additionally, pretreatment with the SB203580 or SP600125 for 1 hour significantly ameliorated the positive effects of 5 μg/mL oxLDL on NO production in EPCs (Fig 4D). Akt phosphorylation induced by 5 μg/mL of oxLDL was also reduced by SB203580 and SP600125 (Fig 4E). These results suggest that Akt activation contribute to the increased tube formation in EPCs treated with 5 μg/mL oxLDL, which is mediated by the p38 MAPK- and SAPK/JNK-related pathways.

### The NADPH Oxidase-related Pathway Contribute to Decreased Tube Formation in EPCs Treated with High Concentrations of OxLDL

**Fig 5:**
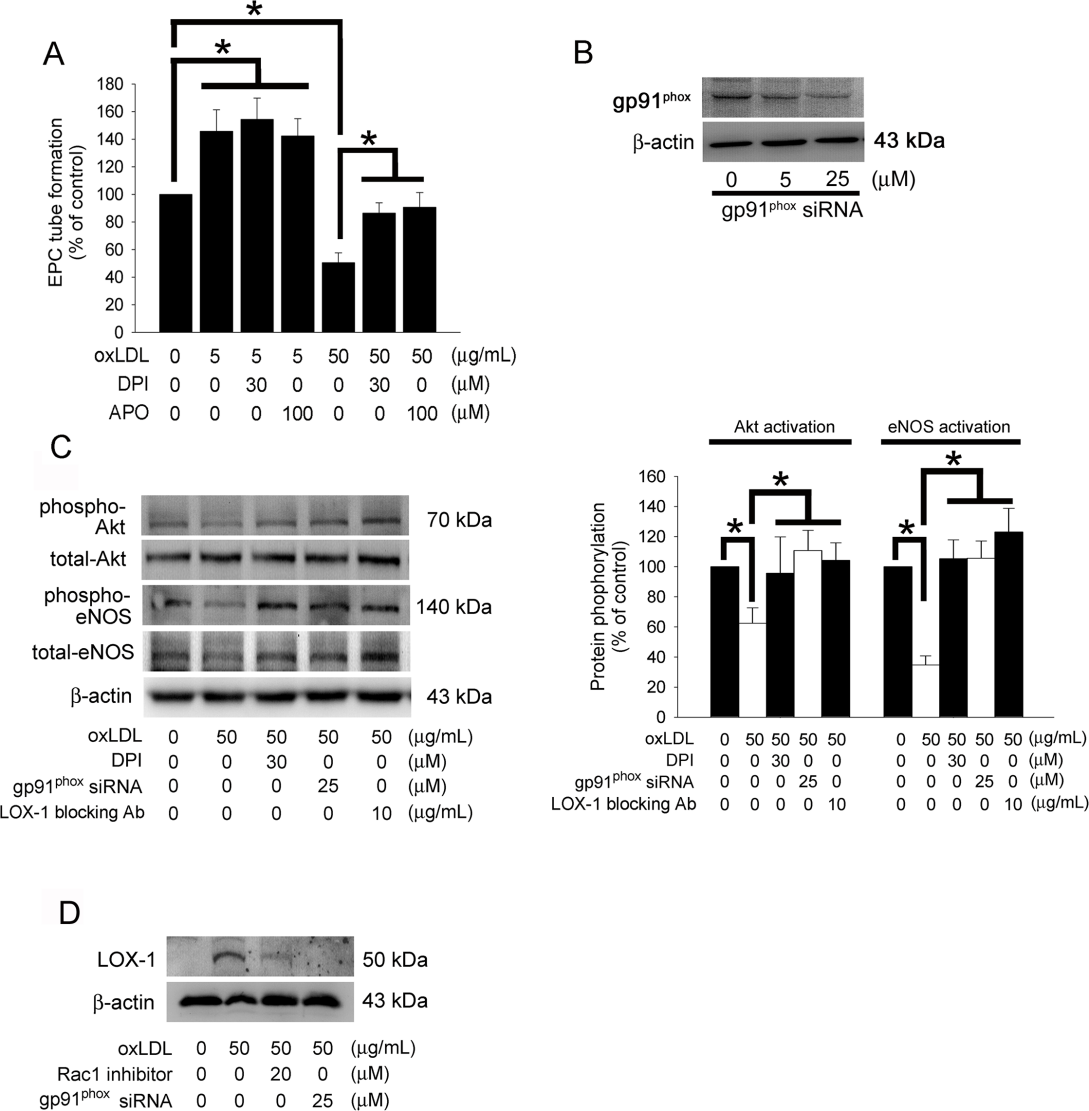
The NADPH oxidase-related pathway contribute to decreased tube formation in EPCs under high concentrated oxLDL. (A) EPCs were pretreated with DPI or APO for 1 hour prior to treatment with 5 or 50 μg/mL oxLDL for 24 hours. *In vitro* angiogenesis was assayed using ECMatrix gel. Data were expressed as the mean ± SEM of three experiments performed in triplicate. *p < 0.05 was considered significant. (B) EPCs were transfected with gp91^phox^ siRNA, the total gp91^phox^ protein were analyzed using western blotting. (C) EPCs were pretreated with DPI, LOX-1 blocking antibody for 1 hour or transfected with gp91^phox^ siRNA prior to 50 μg/mL of oxLDL treatment, subsequently eNOS and Akt activation (phosphorylation) were analyzed by Western blot. Total eNOS, Akt, and β-actin protein levels were used as loading controls. The graph showed the quantitative activation of eNOS (phospho-eNOS/total-eNOS ratio) and Akt (phospho-Akt/total-Akt ratio) density in oxLDL-treated EPCs. (D) EPCs were pretreated with 20 μM Rac1 inhibitor for 1 hour or transfected with gp91^phox^ siRNA prior to 50 μg/mL oxLDL treatment for 12 hours, subsequently membrane LOX-1 expression was analyzed by Western blot. β-actin protein levels were used as a loading control.

In addition, the potential role of NADPH oxidase-mediated oxidative stress in the neovascularization of EPCs affected by oxLDL was investigated using optimal concentrations of diverse antioxidants base on our pilot studies. The specific NADPH oxidase inhibitors (Diphenyleneiodonium, DPI and apocynin, APO) significantly rescued the decreasing of tube formation capacity in EPCs stimulated with 50 μg/mL oxLDL; however, these oxidase inhibitors did not influence EPCs treated with 5 μg/mL oxLDL (Fig 5A). These results suggest that the NADPH–oxidase-derived reactive oxygen species (ROS) signaling pathways are involved in oxLDL-impaired neovascularization in EPCs. NADPH oxidase is a complex enzyme consisting of two membrane-bound components (gp91^phox^ and p22 ^phox^) and three components in the cytosol (Rac 1, p40 ^phox^, p47 ^phox^, and p67 ^phox^). Therefore, gp91^phox^ siRNA and Rac 1 inhibitor were used to block the function of NADPH oxidase in this study. The efficiency of siRNA was showed (Fig 5B). Recent evidence demonstrated that NADPH oxidase-mediated ROS regulates Akt phosphorylation and activation of eNOS [44]. Similarly, our results showed that treatment of DPI or knock down of gp91^phox^ using specific siRNA prior to treatment with 50 μg/mL oxLDL may reverse the inhibition of Akt and eNOS activation (Fig 5C). Additionally, pretreatment with the LOX-1 antibody may block the reduction of Akt and eNOS activation (Fig 5C); gp91^phox^ siRNA transfection and pretreatment with 20 μM of a Rac1 inhibitor for 1 hour prior to oxLDL treatment may efficiently inhibit membrane LOX-1 expression induced by 50 μg/mL oxLDL (Fig 5D). These results suggest that NADPH oxidase plays a significant role in LOX-1 expression mediated by 50 μg/mL oxLDL and the impaired neovascularization mediated by the Akt and eNOS transcriptional regulatory signaling pathway.

### Biphasic Effects of OxLDL on *In Vivo* Neoangiogenesis of Late-outgrowth EPCs

**Fig 6:**
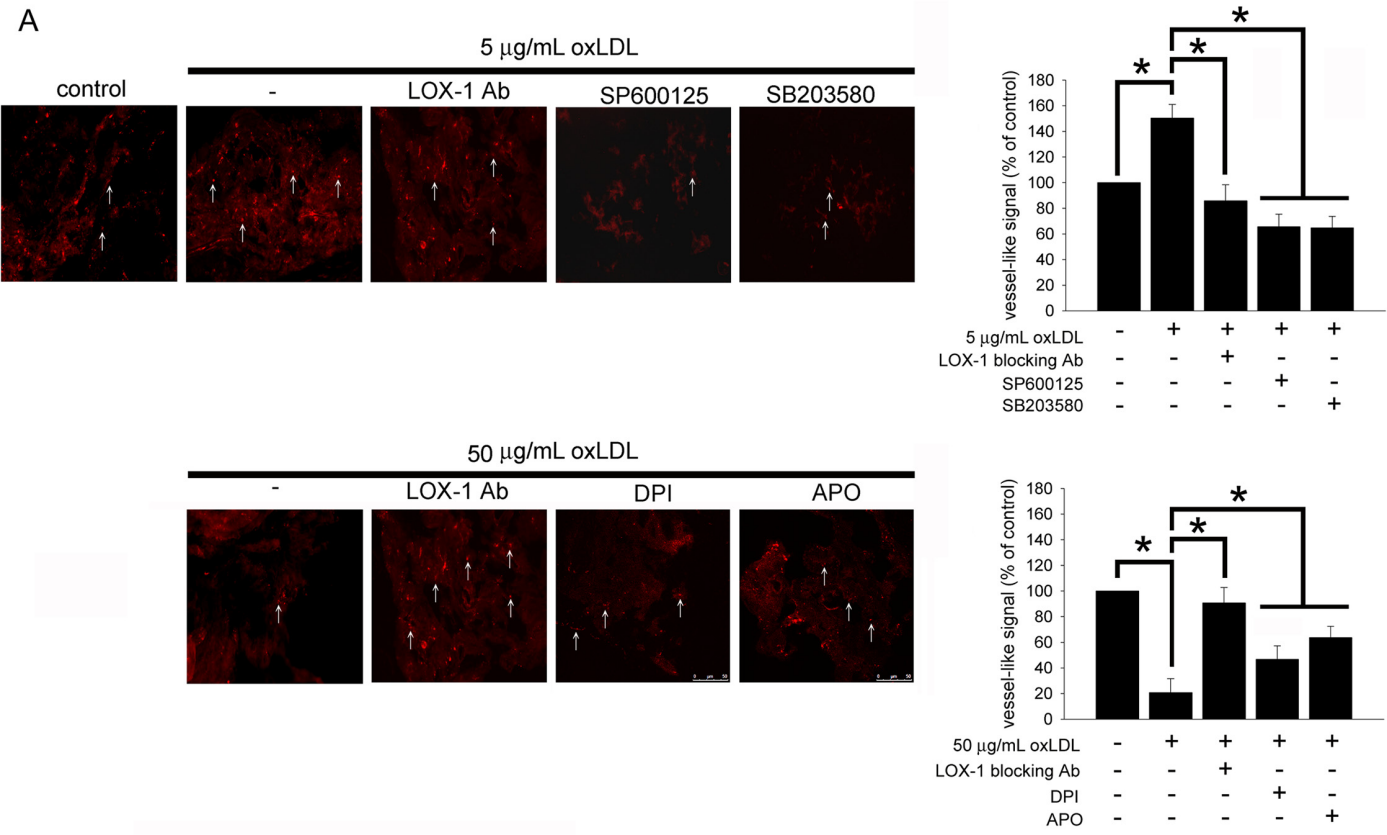
OxLDL affects EPC neovascularization *in vivo*. The vessel networks were stained with anti-human CD31 antibodies and observed using fluorescent microscopy. In the control group, subcutaneous implantation of an EPC/neovascularization gel for 21 days resulted in a small amount of EPC in-growth. Inclusion of 5 μg/mL of oxLDL in the gel significantly increased the formation of vessel-like structures, and LOX-1 blocking antibody, SP600125, and SB203580 against the increased capacity observed in the gel containing 5 μg/mL of oxLDL alone. In contrast, 50 μg/mL of oxLDL in the gel significantly decreased the formation of vessel-like structures, and LOX-1 blocking antibody, DPI, and APO improved the decreased capacity observed in the gel containing 50 μg/mL of oxLDL alone. The vessel-like structures are indicated by white arrows, and all magnifications are at 400x. Data were expressed from one of three experiments. The diagram shows the quantification of vessel-like structures. Data are expressed as the mean ± SEM of three experiments. *p < 0.05 was considered significant.

To investigate the *in vivo* effects of oxLDL on neoangiogenesis, EPC/neoangiogenesis gel constructs were implanted into NOD SCID mice. The constructs were harvested 21 days after implantation and embedded into OCT (frozen tissue matrix). The frozen sections were assessed by immuno-histochemical staining using CD31. The results are presented in Fig 6. Consistent with the *in vitro* results, human EPCs-arranged vessel-like structures were significantly more visible in human EPC/neoangiogenesis gels containing 5 μg/mL of oxLDL in comparison to the control group. In contrast, the raising of human EPCs-arranged vessel-like structures was significantly decreased in EPC/neoangiogenesis gels containing 10 μg/mL of LOX-1 blocking antibody, SP600125, or SB203580 plus 5 μg/mL of oxLDL compared to 5 μg/mL of oxLDL group (Fig 6A). However, adding LOX-1 blocking antibody, DPI or APO to gels reversed the decreasing formation of vessel-like structures in the 50 μg/mL of oxLDL containing gel-received mice.

## Discussion

The schematic shown in S2 Fig summarizes our current results. We demonstrated for the first time that oxLDL has biphasic effects on human late-outgrowth EPCs. OxLDL may regulate EPC vasculogenic function via membrane receptors, LOX-1, and PI3K/Akt- as well as through NO-mediated mechanisms. Low concentrations (approximately 5 μg/mL) of oxLDL enhanced EPC capacity for tube formation in vitro and in vivo by activating eNOS mechanisms, which were mediated by p38 MAPK- and SAPK/JNK-related pathways. Whereas oxLDL at higher concentrations (10–50 μg/mL) impaired EPC function via the activation of NADPH oxidase pathways with consequent inhibition of eNOS activity. eNOS is pivotal for EPC mobilization, migration and vessel formation [45,46]. Our study results demonstrate the diverse EPC vasculogenic function in response to low and high serum oxLDL concentrations.

Previous studies have demonstrated that treating early-EPCs with higher concentrations of oxLDL (10–200μg/mL) may have resulted in p38 MAPK pathway activation [47], decreasing Akt phosphorylation, eNOS mRNA expression and increasing LOX-1 mRNA expression; which could subsequently result in increased early-EPCs apoptosis, decreased early-EPC proliferation, migratory rate and adhesion function, down-regulating E-selectin and integrin α_V_β_5_ (50 μg/mL) expression [27], and impaired therapeutic vasculogenesis [26]. Although our work, similar to previous works, showed the detrimental effects of high oxLDL on early-EPCs, we also demonstrated that late out-growth EPCs were vulnerable to higher concentrations of oxLDL (10–50μg/mL), resulting in impaired EPC tube formation in vivo and in vitro. However, the underlying mechanisms that are involved in late-outgrowth EPC dysfunction under high oxLDL are somewhat different to those in early-EPCs or endothelial cells. In our study, signaling pathways including p38 MAPK, SAPK/JNK, and ERK 1/2 were neither activated nor suppressed and were not involved in the pathogenesis of higher oxLDL conditions.

In our study, the detrimental effect of high oxLDL on EPCs was LOX-1 dependent. Similar to previous studies where treatment of early-EPCs with higher oxLDL concentrations up-regulated cell membrane LOX-1 expression, our study showed that LOX-1 expression of late-outgrowth EPCs was significantly enhanced at oxLDL concentrations higher than 10μg/mL. Interestingly, in our study, oxLDL did not enhance LOX-1 expression at concentrations below 10μg/mL. Unlike other scavenger receptors, such as SR-A nor SR-B, the expression of the LOX-1 receptor, a lectin-like oxLDL receptor that is responsible for the binding and uptake of oxLDL [48], was up-regulated on late-outgrowth EPC cell membrane when treating with higher oxLDL (10–50μg/mL,). In our study, LOX-1 blocking antibody significantly restored the impaired Akt and eNOS activity in higher oxLDL conditions, and also significantly reversed high oxLDL impaired EPC tube formation in a dose-dependent manner.

Oxidative stress plays an important role in the pathogenesis of endothelial dysfunction and cardiovascular disease, especially in cases of dyslipidemia, hypertension, diabetes, smoking and congestive heart failure. Previous studies have suggested that unlike mature endothelial cells, EPCs are more resistant to ROS-driven cytotoxicity because EPCs may play a role in repairing injured endothelium or are involved in neo-vasculogenesis in ischemic tissue enriched for reactive oxygen species and oxidized metabolites. It has been known that activation of LOX-1 can stimulate the formation of reactive oxygen species (ROS) and initiate a series of redox-sensitive signaling pathways. In our study, the up-regulation of the LOX-1 receptor by higher concentrations of oxLDL revealed the possible role of ROS regulation on EPC functions. The lack of enhancement of LOX-1 expression by lower concentrations of oxLDL in our study implies that ROS was not significantly activated in these situations. Under higher concentrations of oxLDL (10–50 μg/mL), the up-regulated LOX-1 receptors subsequently potentiate the activation of NADPH oxidase and further impair Akt activation; eNOS phosphorylation and eventually EPC tube formation. The detrimental effects of higher oxLDL could be reversed by the anti-oxidants: DPI, Apocynin and gp91^phox^ siRNA. Our study corroborated a previous study demonstrating that EPCs are not completely oxidative stress-resistant, and NADH/NADPH oxidase was suggested to be one of the most important enzyme systems capable of producing ROS in the endothelial system [49].

Previous studies showed that LOX-1 receptors could be up-regulated by oxLDL treatment [50] or oxidative stress. In our study, higher rather than lower concentrations of oxLDL enhanced LOX-1 expression. Rac1 inhibitors and gp91^phox^ siRNA abrogated the expression of LOX-1 induced by higher oxLDL concentrations. Increased LOX-1 expression could have created more oxidative stress, and this oxidative stress could have further enhanced the expression of LOX-1, which could form a positive feedback loop maintaining the detrimental effect of higher oxLDL concentrations on EPCs.

We first noted that in contrast to higher oxLDL concentrations, treating EPCs with lower oxLDL (0.1 to 5 μg/mL) concentrations, corresponding to those in serum levels of relatively healthy populations, could paradoxically enhance EPC survival and tube formation in vivo and in vitro. The beneficial effect of lower oxLDL concentrations was also LOX-1 dependent and not via SR-A or SR-B1. After LOX-1 receptors on EPCs were activated by lower concentrations of oxLDL, the p38/MAPK and SAPK/JNK pathways were subsequently activated and further enhanced Akt-eNOS pathway activation and EPC tube formation. Although the beneficial effect of low oxLDL has been demonstrated in previous endothelium studies, the underlying mechanisms are quite different and were not redox-dependent for EPCs.

In our study, anti-oxidants used with DPI and Aponcynin failed to inhibit EPC tube formation augmented by lower oxLDL concentrations, suggesting that oxidative stress is not involved in the mechanisms that promote EPC tube formation by lower oxLDL concentrations. In a previous study conducted by Dandapat et al. focusing on mature endothelial cells, it was demonstrated that small concentrations of oxLDL (0.1 to 5 μg/mL) could enhance endothelium tube formation by inducing the expression of the Vascular endothelial growth factor via LOX-1 mediated activation of NADPH oxidase- MAPKs-NF-κB pathways. According to current studies, the mechanisms resulting in the discrepancies between endothelium and EPCs under lower concentrations of oxLDL is still not known. However, some possible clues may answer this question. In our study, although lower oxLDL (0.1–5 μg/mL) concentrations exert positive effects via the LOX-1 receptor, the amounts of lectin-like oxLDL receptors were not up-regulated until oxLDL concentrations were above 10 μg/mL (at which the detrimental effects of oxLDL started). In contrast to our study, LOX-1 in mature endothelium was significantly enhanced dose-dependently even in very low concentrations of oxLDL (0.1–5 μg/mL) [51], which may further activate NADPH oxidase in endothelium. In addition, previous studies have demonstrated that EPCs are more redox resistance under some circumstances and may have special machinery to clear oxidative stress and may explain why lower concentrations of oxLDL failed to generate NADPH oxidase, which could possibly reverse the enhanced LOX-1 expression, in EPCs. Further studies could be designed to examine this idea about oxidative scavengers in EPCs treated with lower concentrations of oxLDL.

In conclusion, our study first demonstrated that oxLDL is not always harmful to EPCs. We demonstrated that oxLDL has concentration-dependent bi-phasic effects on late-outgrowth EPC tube formation in vitro and in vivo. Further animal and prospective clinical studies could be initiated to delineate the clinical implications of the concentration-dependent effects of serum oxLDL on the neo-vasculogenic function of EPCs.

## Supporting Information

S1 Fig The late EPC-derived EC outgrowth population was also characterized. The late EPC-derived EC outgrowth population was also characterized using immunofluorescent staining for the expression of lectin, VE-cadherin, von Willebrand factor (vWF), CD31 (PE-CAM), CD34, kinase insert domain receptor (KDR)/VEGF receptor 2, CD133 and the endocytic portion of Dil-acLDL. The vascular smooth muscle cell markers (αSMA, CALP, SM-MHC) and leukocyte marker (CD45) were undetectable.
(TIF)

S2 Fig The schematic shows summarize of our current results. OxLDL has biphasic effects on human late-outgrowth EPCs. OxLDL may regulate EPC vasculogenic function via membrane receptors, LOX-1, and PI3K/Akt- as well as through NO-mediated mechanisms. Low concentrations (approximately 5 μg/mL) of oxLDL enhanced EPC capacity for tube formation in vitro and in vivo by activating eNOS mechanisms, which were mediated by p38 MAPK- and SAPK/JNK-related pathways. Whereas oxLDL at higher concentrations (10–50 μg/mL) impaired EPC function via the activation of NADPH oxidase pathways with consequent inhibition of eNOS activity.
(TIF)

S1 Text (DOC)
