# Supplementary figures and images for: The Biphasic Effects of Oxidized-Low Density Lipoprotein on the Vasculogenic Function of Endothelial Progenitor Cells
Source: PLoS One. 2015 May 27;10(5):e0123971. doi: 10.1371/journal.pone.0123971 (PMC4446352; doi:10.1371/journal.pone.0123971)

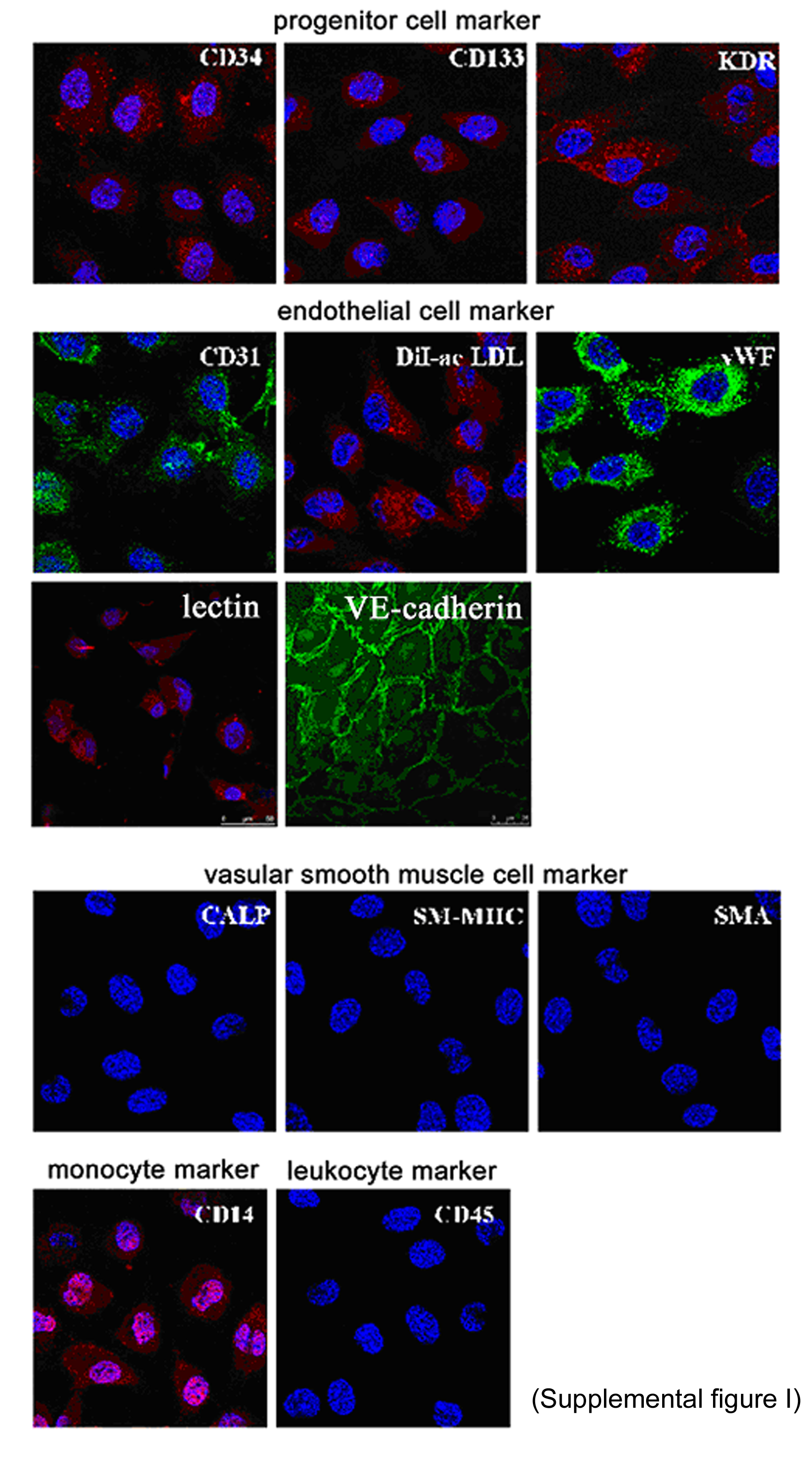

Supplement: S1 Fig — The late EPC-derived EC outgrowth population was also characterized using immunofluorescent staining for the expression of lectin, VE-cadherin, von Willebrand factor (vWF), CD31 (PE-CAM), CD34, kinase insert domain receptor (KDR)/VEGF receptor 2, CD133 and the endocytic portion of Dil-acLDL. The vascular smooth muscle cell markers (αSMA, CALP, SM-MHC) and leukocyte marker (CD45) were undetectable. (TIF) [file pone.0123971.s001.tif]

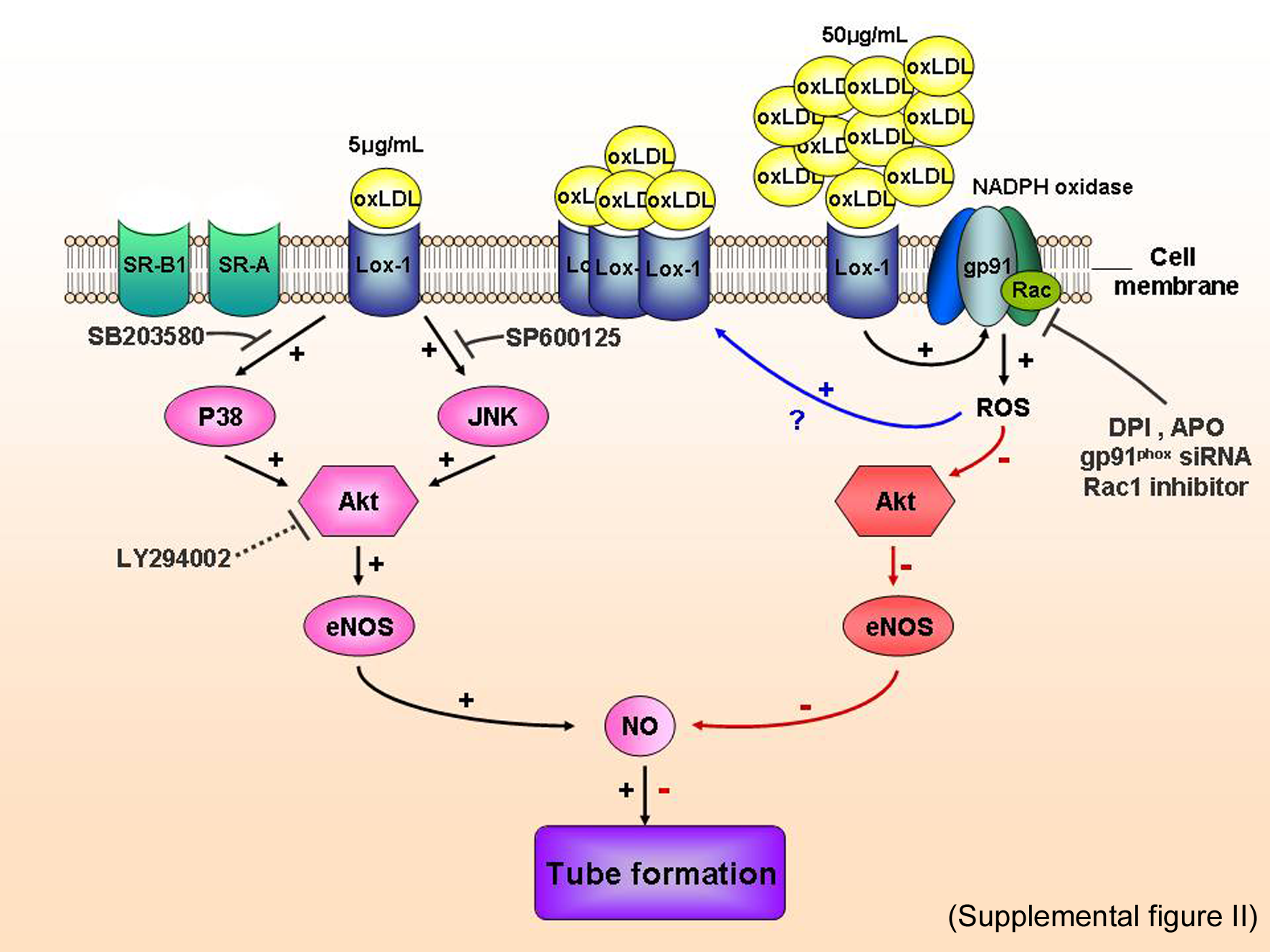

Supplement: S2 Fig — OxLDL has biphasic effects on human late-outgrowth EPCs. OxLDL may regulate EPC vasculogenic function via membrane receptors, LOX-1, and PI3K/Akt- as well as through NO-mediated mechanisms. Low concentrations (approximately 5 μg/mL) of oxLDL enhanced EPC capacity for tube formation in vitro and in vivo by activating eNOS mechanisms, which were mediated by p38 MAPK- and SAPK/JNK-related pathways. Whereas oxLDL at higher concentrations (10–50 μg/mL) impaired EPC function via the activation of NADPH oxidase pathways with consequent inhibition of eNOS activity. (TIF) [file pone.0123971.s002.tif]
