# Supplementary material for: The Biphasic Effects of Oxidized-Low Density Lipoprotein on the Vasculogenic Function of Endothelial Progenitor Cells
Source: PLoS One. 2015 May 27;10(5):e0123971. doi: 10.1371/journal.pone.0123971 (PMC4446352; doi:10.1371/journal.pone.0123971)
Supplement: S1 Text — (DOC) [file pone.0123971.s003.doc]

**Supplementary Information**

The Biphasic Effects of Oxidized-Low Density Lipoprotein on the Vasculogenic Function of Endothelial Progenitor Cells

**Discussion**

According to previous clinical studies, the physiological serum concentrations of healthy subjects were at around 5.7-14.8 μg/ml; and at around 18.8 -20.2 μg/ml in human beings who had diabetes or coronary artery disease . However, to the best of our knowledge, the concentrations of oxLDL in other tissue are not accessible now. We have provided this information in the introduction section of our manuscript already. Since EPCs were mobilized from bone marrow, circulating in the blood stream and landing on endothelial surface preparing for homing process, the rational to treat EPCs in our study by the concentrations corresponding to serum levels should be reasonable. Just similar to the major view-points of previous studies on oxLDL, which was harmful; our study demonstrated that high concentrations of oxLDL impaired EPC tube formation. However, we also demonstrated that oxLDL at low concentrations in reverse enhanced EPC tube formation, which corresponds to a few studies showing that low concentrations of oxLDL may paradoxically protect mature endothelial cells against apoptosis and even more, enhance tube formation . Although we had demonstrated the bi-phasic effects of oxLDL on EPC tube formation in this study, however it is still not enough to tell a comprehensive physiological means of the biphasic phenomenon only based on these findings. There are several limitations: First, among the various kinds of lipoproteins, not only oxLDL, but also HDL and other lipoproteins could also influence vasculogenesis , the interactions effects on vasculogenesis between different lipoprotein should be considered . Second, although angiogenesis may enhance the healing process of ischemic tissue , however, when considering atherosclerosis plaque stability, enhancing angiogenesis may have deleterious effects by causing plaque expansion and vulnerability, and further intravascular thrombosis .

**References**

1. Ross R (1999) Atherosclerosis--an inflammatory disease. N Engl J Med 340: 115-126.

2. Isomaa B, Almgren P, Tuomi T, Forsen B, Lahti K, et al. (2001) Cardiovascular morbidity and mortality associated with the metabolic syndrome. Diabetes Care 24: 683-689.

3. Trevisan M, Liu J, Bahsas FB, Menotti A (1998) Syndrome X and mortality: a population-based study. Risk Factor and Life Expectancy Research Group. Am J Epidemiol 148: 958-966.

4. van der Zwan LP, Teerlink T, Dekker JM, Henry RM, Stehouwer CD, et al. (2009) Circulating oxidized LDL: determinants and association with brachial flow-mediated dilation. J Lipid Res 50: 342-349.

5. Calmarza P, Trejo JM, Lapresta C, Lopez P (2014) LDL oxidation and its association with carotid artery intima-media thickness and other cardiovascular risk factors in a sample of Spanish general population. Angiology 65: 357-362.

6. Koenig W, Karakas M, Zierer A, Herder C, Baumert J, et al. (2011) Oxidized LDL and the risk of coronary heart disease: results from the MONICA/KORA Augsburg Study. Clin Chem 57: 1196-1200.

7. Moellering DR, Levonen AL, Go YM, Patel RP, Dickinson DA, et al. (2002) Induction of glutathione synthesis by oxidized low-density lipoprotein and 1-palmitoyl-2-arachidonyl phosphatidylcholine: protection against quinone-mediated oxidative stress. Biochem J 362: 51-59.

8. Dandapat A, Hu C, Sun L, Mehta JL (2007) Small concentrations of oxLDL induce capillary tube formation from endothelial cells via LOX-1-dependent redox-sensitive pathway. Arterioscler Thromb Vasc Biol 27: 2435-2442.

9. Huang CY, Lin FY, Shih CM, Au HK, Chang YJ, et al. (2012) Moderate to high concentrations of high-density lipoprotein from healthy subjects paradoxically impair human endothelial progenitor cells and related angiogenesis by activating Rho-associated kinase pathways. Arterioscler Thromb Vasc Biol 32: 2405-2417.

10. Isner JM, Losordo DW (1999) Therapeutic angiogenesis for heart failure. Nat Med 5: 491-492.

11. Kahlon R, Shapero J, Gotlieb AI (1992) Angiogenesis in atherosclerosis. Can J Cardiol 8: 60-64.

12. Bochkov VN, Philippova M, Oskolkova O, Kadl A, Furnkranz A, et al. (2006) Oxidized phospholipids stimulate angiogenesis via autocrine mechanisms, implicating a novel role for lipid oxidation in the evolution of atherosclerotic lesions. Circ Res 99: 900-908.
